# Overexpression of Mesothelin in Pancreatic Ductal Adenocarcinoma (PDAC)

**DOI:** 10.7150/ijms.39012

**Published:** 2020-02-04

**Authors:** Kai Le, Jia Wang, Tao Zhang, Yifan Guo, Hong Chang, Siyuan Wang, Bin Zhu

**Affiliations:** 1 Department of General Surgery, Peking University Ninth School of Clinical Medicine, Beijing Shijitan Hospital, Capital Medical University, Beijing, China; 2 Department of Urology Surgery, Aerospace Center Hospital, Beijing, China; 3 Department of General Surgery, Liang Xiang Teaching Hospital of Capital Medical University, Beijing, China; 4 Department of Pathology, Beijing Shijitan Hospital, Capital Medical University, Beijing, China; 5 Department of Rehabilitation Medicine, China-Japan Friendship Hospital, Beijing, China

**Keywords:** pancreatic ductal adenocarcinoma (PDAC), mesothelin (MSLN), anti-mesothelin antibodies, immunohistochemistry (IHC)

## Abstract

**Purpose**: Pancreatic ductal adenocarcinoma (PDAC) with difficulty in early diagnosis does not respond well to conventional treatments and has not occurred significant improvement in the overall 5-year survival rates. Mesothelin (MSLN) is a tumor differentiation antigen expressed in several solid neoplasms and a limited number of healthy tissues. Its selective expression on malignant cells makes it an interesting candidate for investigation as a diagnostic and prognostic biomarker and as a therapeutic target. In this study, we detected the expression of MSLN in PDAC and analyzed the correlation between the expression of MSLN and clinicopathological data, so as to provide more theoretical basis for the role of MSLN in the diagnosis and treatment of PDAC.

**Patients and methods**: Cancer and para-cancer tissues of 24 cases with PDAC were assessed by standardized immunohistochemical (IHC) detection with two kinds of anti-MSLN antibodies (EPR4509 and EPR19025-42) to detect their positive expression rates and study the correlation between the expression of MSLN and the clinicopathological data.

**Results**: The two anti-MSLN antibodies of cancer tissues showed positive expression with tan yellow or tan brown granules diffusely distributed on the cell membrane in 22 of 24 cases with PDAC (positive rate of 91.67%), and the positive expression of the two antibodies EPR4509 and EPR19025-42 was completely consistent in all tissue samples. No expression of the two anti-MSLN antibodies was found in para-cancer tissues and the difference was statistically significant (χ^2^=40.615, p=0.000, p<0.05) when compared with PDAC tissues. There was no significant correlation between MSLN expression and clinicopathological data, such as gender, tumor size, location, pathological stage, differentiation degree and lymph node metastasis (p>0.05).

**Conclusion**: MSLN was highly expressed in PDAC tissues, but not in paracancerous tissues. There was no significant correlation between MSLN expression and clinicopathological factors. The overexpression of MSLN may have promising prospects in diagnosis, targeted therapy and immunotherapy of PDAC.

## Introduction

Pancreatic cancer (PC) is a malignant tumor of the digestive system with poor prognosis. PC usually refers to pancreatic ductal adenocarcinoma (PDAC) which is the most common type of PCs. It is predicted that PC will become the second leading cause of cancer-related mortality worldwide in the next decade.^1^-^4^ In recent years, the morbidity and mortality of PC have the trend of growth. It was estimated that by 2020, the incidence of PC would be as high as 420,000, and the number of related deaths would be about 410,000.^5^ Despite the improvements in therapy, the overall 5-year survival rates for PC remained relatively unchanged. PC remains a disease that does not respond well to surgery, chemotherapy, or radiotherapy. Difficulties in understanding the complex genetics of tumors, metabolic changes, complex interactions of PC cells with stromal cells, immune cells, and endothelial cells contribute to the poor overall therapeutic effects of anti-cancer therapy in patients with PC.^6^

Mesothelin (MSLN) is a tumor-associated antigen firstly discovered in 1992.^2^,^7^ It is expressed in a limited number of healthy tissues, including the pleura, peritoneum, pericardium and epithelium of the trachea, but is highly expressed in several types of solid neoplasms, including malignant mesothelioma, ovarian cancer and PDAC.^8^,^9^ Previous studies have found that the overexpression of MSLN has implications to cancer development and progresssion.^10^,^11^ Its selective expression on malignant cells has made it an interesting candidate for investigation as a diagnostic and prognostic biomarker and as a therapeutic target. 2,10

In this study, two independent anti- MSLN antibodies were used to evaluate the positive expression rate of cancer tissues and para-cancer tissues in 24 cases with PDAC by standardized manual immunohistochemical (IHC) detection method, and the correlation between MSLN expression and clinicopathological data was analyzed.

## Material and methods

### Patients and tissue samples

We collected paraffin tissues from 24 PDAC cases. Samples were from Department of Pathology, Beijing Shijitan Hospital, Capital Medical University, Beijing, China. All cancer and para-cancer tissue samples were obtained from radical operation of PC from 2013 to 2018. Complete clinical data (age, gender, tumor size, location, pathological type, lymphatic metastasis, differentiation degree and TNM stage) were recorded. The current study was approved by the local ethics committee. Written informed consent was obtained from the participants. These patients have not been treated with chemotherapy, radiotherapy and immunotherapy.

### Monoclonal antibodies

Anti-MSLN antibodies which are “Rabbit monoclonal (EPR4509, ab133489) to Mesothelin” and “Rabbit monoclonal (EPR19025-42, ab196235) to Mesothelin” were purchased from Abcam.^12^ Antibodies were diluted 1:200 for EPR4509 and 1:2000 for EPR19025-42.

### Immunohistochemistry (IHC)

All paraffin specimens were independently evaluated using a standardized manual IHC detection system. Antigen retrieval was performed for 3 minutes using an autoclave with citrate buffer (pH 6.0). Sections were probed with anti-MSLN antibodies in a humid chamber for 4 hours at room temperature and labeled using an EnVision Assay Kit (Dako) for 30 minutes without a signal amplification system. IHC of MSLN was assessed using a revised grading standard based on the scoring systems shown in Tables 1 & 2. IHC scores were performed by three pathologists without the need to predict FISH results. Three independent pathologists were not participating in the study program and were blinded to the group of the samples. The mean value of three pathologists was target score.

### Evaluation standards

The target score was obtained from the addition of Scores (1) + (2). Positivity of MSLN was defined as a target score greater than three, or strong MSLN staining (as shown in Tables 1 & 2).^13^-^15^

### Statistical analysis

All statistical analyses were performed using SPSS (Statistical Product and Service Solutions, IBM) software (version 21.0). Mean ± standard deviation was used for quantitative data. Data analysis was performed using the Chi-square test (Fisher's exact test) by professional statistician. A p-value < 0.05 was considered statistically significant.

## Results

**Figure 1:**
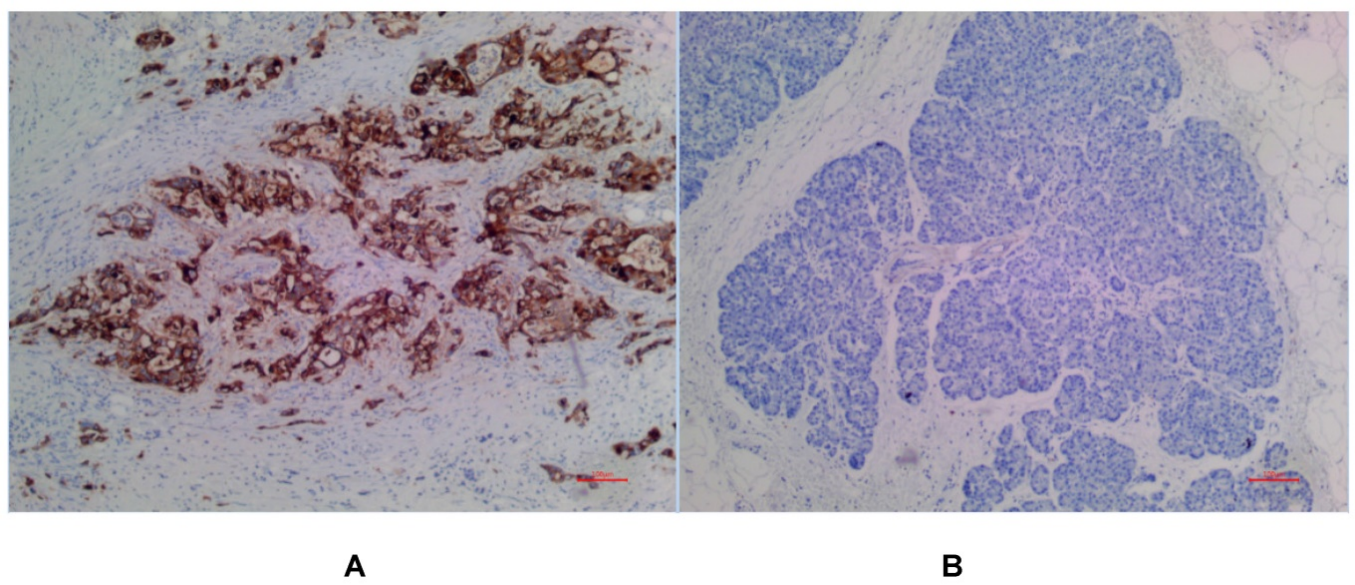
(A) Positive expression of MSLN in PDAC for EPR4509. (B) No expression of MSLN in para-cancer tissue for EPR4509.

**Figure 2:**
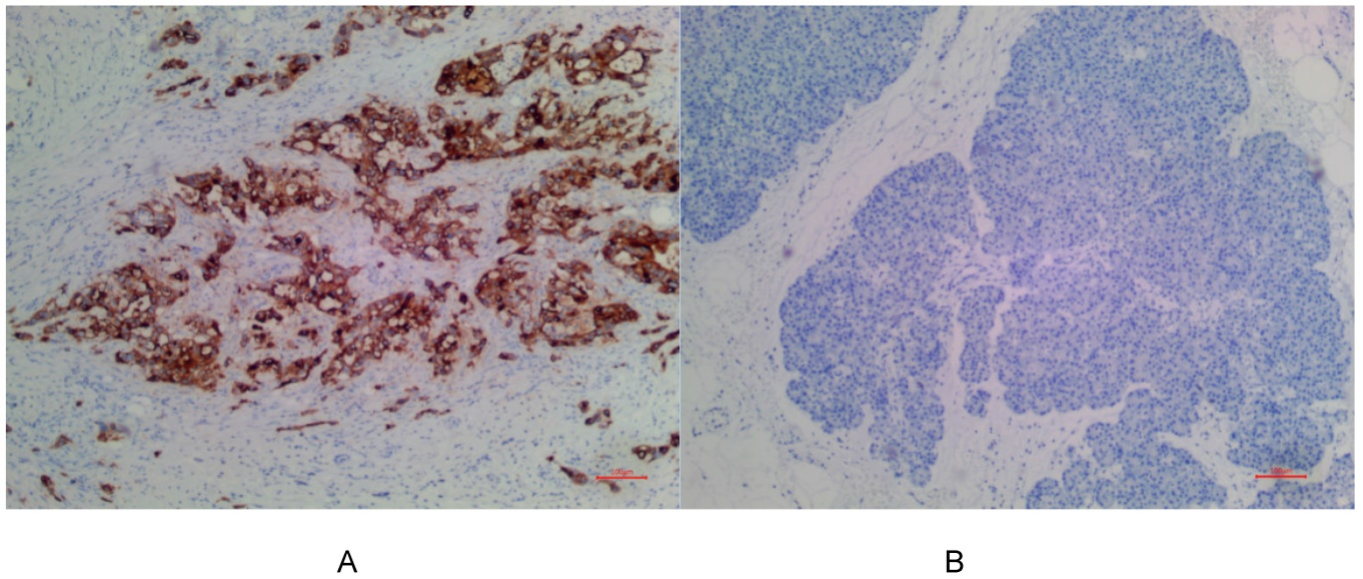
(A) Positive expression of MSLN in PDAC for EPR19025-42. (B) No expression of MSLN in para-cancer tissue for EPR19025-42.

**Figure 3:**
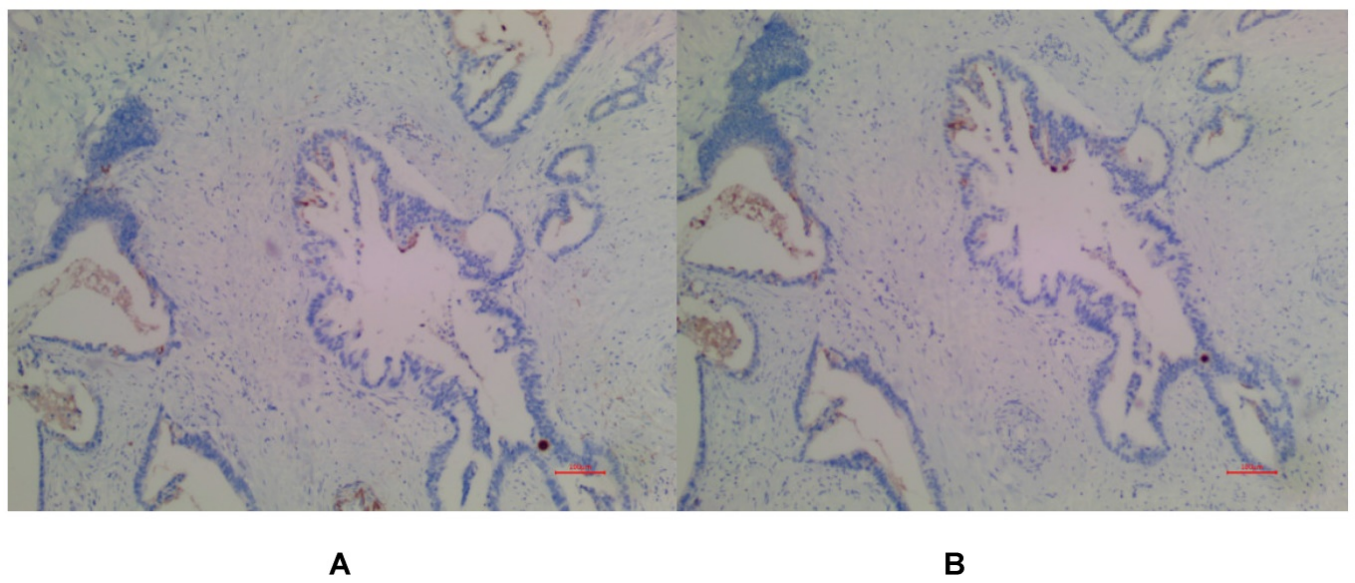
(A) Negative expression of MSLN in PDAC for EPR4509. (B) Negative expression of MSLN in PDAC for EPR19025-42.

In PDAC tissues, MSLN was diffusely distributed on the cell membrane, with tan yellow or tan brown granules. Among the 24 samples of PDAC, 22 of the two antibodies EPR4509 and EPR19025-42 showed positive MSLN, with a positive expression rate of 91.67% (22/24), and the positive expression of the two antibodies was completely consistent in all tissue samples. No expression of MSLN was found in para-cancer tissues of the two antibodies, and the difference was statistically significant (χ^2^=40.615, p=0.000) (as shown in Fig. 1, 2 and 3). Of the cases, 95.45% (21/22) for EPR4509 and 90.91% (20/22) for EPR19025-42 showed MSLN positivity in over 50% of cells. Of these cases, 63.64% (14/22) for EPR4509 and 59.09% (13/22) for EPR19025-42 displayed strong MSLN staining. Approximately 59.09% (13/22) for EPR4509 and 50.00% (11/22) for EPR19025-42 had strong MSLN staining in over 50% of cells. When compared to PDAC, para-cancer tissues showed no MSLN expression using either EPR4509 or EPR19025-42 (p<0.05). There was no significant correlation between MSLN expression and clinicopathological factors, such as gender, tumor size, location, pathological stage, differentiation degree and lymph node metastasis(p>0.05) (as shown in Table 3).

## Discussion

PC is characterized by occult clinical manifestations, difficulty in early diagnosis, rapid progression and prone to distant metastasis.^1^,^3^,^4^ PC has become one of the major malignant tumors that seriously endanger human life and health.^1^-^4^ MSLN is a cell-surface protein expressed in a limited number of healthy tissues, but is highly expressed in several types of solid tumors, including malignant mesothelioma, lung adenocarcinoma, ovarian cancer, and PDAC.^16^-^20^ In PC, MSLN has been investigated mainly to address two unmet issues: the urgent need for new methods for early diagnosis and the lack of successfully targetable oncogenic alterations for patients' treatment.^2^,^10^,^11^ Up to now, several studies have evaluated the sensitivity, specificity, and accuracy of MSLN for PDAC diagnosis both on cytologic and histologic pancreatic specimens. Overall, MSLN achieved a 75% sensitivity, 80% specificity, and a diagnostic accuracy of 77% in predicting diagnosis of PDAC. In 2014, the Papanicolaou society of cytopathology included MSLN staining as a supportive tool for PDAC diagnosis. 21

Regarding MSLN expression in PDAC, the first evidence was provided in 2001, Argani et al. found that MSLN mRNA expression was detected in four of four (100%) resected primary PDAC by in situ hybridization, as well as in 18 of 20 (90%) PDAC cell lines by reverse transcription polymerase chain reaction (RTPCR). 22 Inaguma et al. reported that the positive rates of two MSLN antibodies MN-1 and 5B-2 in the tissues of PDAC were 87% and 71% respectively.^23^ These lower detection rates may be related to the sensitivity and specificity of different MSLN antibodies used. Different antibodies recognize different epitopes and have different affinity with MSLN. In the same study, MSLN immunoreactivity was observed in malignant mesothelioma (78% vs 75%), uterine endometrioid adenocarcinoma (63% vs 62%), intrahepatic cholangiocellular carcinoma (41% for both), and invasive ductal carcinoma of the mammary gland (11% vs 12%). In our study, we utilized two independent antibodies for MSLN detection in PDAC. It is the first assessment of MSLN in PDAC using EPR4509 and EPR19025-42 clinically. Both anti-MSLN antibodies provided excellent signal quality. Our results showed high-levels (91.67%) of MSLN expression in PDAC tissues and no MSLN expression in para-cancer tissues. The positive expression of the two antibodies was completely consistent in all tissue samples.This indicates that PDAC has a higher positive MSLN expression rates than other solid tumors, which is related to different levels of methylation of MSLN epigenetic genes in different malignant tumors.^23^ Jhala and coworkers detected MSLN positive staining only in adenocarcinoma cells and not in reactive chronic pancreatitis cells (62% vs 0%) in 65 samples from pancreatic lesions.^24^ These studies may be helpful in differentiating chronic pancreatitis from PDAC through MSLN assessment. At the same time, IHC staining of MSLN can also be used to identify benign or malignant pancreatic ductal epithelial cells.^22^

Argani et al considered that the lower the differentiation degree of PDAC, the higher the expression of MSLN^22^. The positive rate of MSLN in moderately and poorly differentiated PDAC tissues was significantly higher than that in highly differentiated PDAC tissues, suggesting that MSLN may be closely related to the biological behavior of PDAC. Research showed that the expression of MSLN could promote the proliferation and metastasis of PDAC cells, and was closely related to lymph node metastasis. Overexpression of MSLN could significantly increase the volume of tumors in mice and enhance the proliferation and migration ability of PDAC cells.^25^ However, our results showed that MSLN expression in PDAC tissue was not correlated with gender, tumor size, location, pathological stage, differentiation degree and lymph node metastasis, which is probably related to small sample. Of course, if the expression of MSLN in PDAC tissue is not correlated with the clinicopathological data, it is also possible that all PDAC patients with positive expression of MSLN can be targeted and immunotherapy related to MSLN without considering the patient's pathological stage, differentiation degree and lymph node metastasis.

This differential expression may be more helpful for MSLN as a potential target for early diagnosis, targeting and immunotherapy of PDAC. Over the past years, several strategies have been investigated. They include the use of monoclonal antibodies against MSLN or protein carrying toxins or cytotoxic agents, chimeric T cells containing variable fragments that recognize MSLN, and specific vaccines that can induce T-cell immune response against MSLN.^2^,^26^,^27^ In patient-derived xenograft (PDX) models, Jiang and coworkers revealed that anti-MSLN CAR-T-cells could infiltrate PDAC and lead to tumor cell death, using human anti-MSLN antibodies P1A6E and P3F2.^28^ Similarly, in an engineered model of autochthonous PDAC, Stromne's and colleagues demonstrated that the engineered T-cells could suppress tumor growth in the absence of toxicity.^29^ These remarkable results have prompted research into this approach for PDAC.^30^-^38^ MSLN-based therapeutic strategies have obtained scant results in PDAC so far.^28^,^39^ Based on the rationale ensuing from preclinical work, it is hoped that MSLN-based therapy combined with other strategies, such as chemotherapy, targeted therapy and immunotherapy, will improve patient survival.^2^

## Conclusion

MSLN was highly expressed on the cell membrane of PDAC tissues. No expression of MSLN was found in para-cancer tissues. The positive expression of the two antibodies EPR4509 and EPR19025-42 was completely consistent in all tissue samples. There was no significant correlation between MSLN expression and clinicopathological factors, such as gender, tumor size, location, pathological stage, differentiation degree and lymph node metastasis. The expression level of MSLN may have promising prospects in diagnosis, targeted therapy and immunotherapy of PDAC.

## Figures and Tables

**Table 1:** Score (1) was determined from the percentage of stained tumor cells

Percentage of stained tumor cells	Score 1
0%	0
1-10%	1
11-50%	2
51-100%	3

**Table 2:** Score (2) was determined through the assessment of MSLN staining intensity

Staining intensity	Score (2)
No staining	0
Weak	1
Moderate	1
Strong	2

**Table 3:** Correlation between expression of MSLN and clinicopathologic characteristics

Clinical Pathology	Cases		χ^2^	*p*
	Positive 22 (91.67%)	Negative 2 (8.33%)		
**Gender**				
Male	12	2	1.558	0.493
Female	10	0		
**Depth of invasion**				
T1	2	0	1.454	0.594
T2	8	0		
T3	12	2		
**Lymph node metastasis**				
Yes	13	1	0.062	1.000
No	9	1		
**Pathological stage**				
I	2	0	1.866	0.507
II	16	1		
III	4	1		
**Location**				
Head of pancreas	8	1	0.145	1.000
Body and tail of pancreas	14	1		
**Differentiation degree**				
High differentiation	0	0	2.805	0.239
Medium differentiation	20	1		
Low differentiation	2	1		

## Abbreviations

PDAC: pancreatic ductal adenocarcinoma
MSLN: mesothelin
IHC: immunohistochemistry

## References

[1] He X, Li Y, Su T (2018). The impact of a history of cancer on pancreatic ductal adenocarcinoma survival. United European Gastroenterol J.

[2] Nichetti F, Marra A, Corti F (2018). The role of Mesothelin as a diagnostic and therapeutic target in pancreatic ductal adenocarcinoma: A comprehensive review. Target Oncol.

[3] Rahib L, Smith BD, Aizenberg R (2014). Projecting cancer incidence and deaths to 2030: the unexpected burden of thyroid, liver, and pancreas cancers in the United States. Cancer Res.

[4] Scarton L, Yoon S, Oh S (2018). Pancreatic cancer related health disparities: A commentary. Cancers (Basel).

[5] Ferlay J, Soerjomataram I, Dikshit R (2015). Cancer incidence and mortality worldwide: sources, methods and major patterns in GLOBOCAN 2012. Int J Cancer.

[6] Neoptolemos JP, Kleeff J, Michl P (2018). Therapeutic developments in pancreatic cancer: current and future perspectives. Nat Rev Gastroenterol Hepatol.

[7] Chang K, Pai LH, Batra JK (1992). Characterization of the antigen (CAK1) recognized by monoclonal antibody K1 present on ovarian cancers and normal mesothelium. Cancer Res.

[8] Chang K, Pastan I (1996). Molecular cloning of mesothelin, a differentiation antigen present on mesothelium, mesotheliomas, and ovarian cancers. Proc Natl Acad Sci USA.

[9] Urwin D, Lake RA (2000). Structure of the Mesothelin/MPF gene and characterization of its promoter. Mol Cell Biol Res Commun.

[10] Hassan R, Thomas A, Alewine C (2016). Mesothelin immunotherapy for cancer: ready for prime time?. J Clin Oncol.

[11] Yamaguchi N, Hattori K, Oh-eda M (1994). A novel cytokine exhibiting megakaryocyte potentiating activity from a human pancreatic tumor cell line HPC-Y5. J Biol Chem.

[12] Akbari S, Abou-Arkoub R, Sun S (2017). Microparticle formation in peritoneal dialysis: A proof of concept study. Can J Kidney Health Dis.

[13] Bharadwaj U, Li M, Chen C (2008). Mesothelin-induced pancreatic cancer cell proliferation involves alteration of cyclin E via activation of signal transducer and activator of transcription protein 3. Mol Cancer Res.

[14] Rump A, Morikawa Y, Tanaka M (2004). Binding of ovarian cancer antigen CA125/MUC16 to mesothelin mediates cell adhesion. J Biol Chem.

[15] Zheng C, Jia W, Tang Y (2012). Mesothelin regulates growth and apoptosis in pancreatic cancer cells through p53-dependent and-independent signal pathway. J Exp Clin Cancer Res.

[16] Kaneko O, Gong L, Zhang J (2008). A binding domain on mesothelin for CA125/MUC16. J Biol Chem.

[17] Bharadwaj U, Marin-Muller C, Li M (2011). Mesothelin overexpression promotes autocrine IL-6/sIL-6R trans-signaling to stimulate pancreatic cancer cell proliferation. Carcinogenesis.

[18] Chang MC, Chen CA, Hsieh CY (2009). Mesothelin inhibits paclitaxel-induced apoptosis through the PI3K pathway. Biochem J.

[19] Servais EL, Colovos C, Rodriguez LA (2012). Mesothelin overexpression promotes mesothelioma cell invasion and MMP-9 secretion in an orthotopic mouse model and in epithelioid pleural mesothelioma patients. Clin Cancer Res.

[20] Hollevoet K, Nackaerts K, Gosselin R (2011). Soluble mesothelin, megakaryocyte potentiating factor, and osteopontin as markers of patient response and outcome in mesothelioma. J Thorac Oncol.

[21] Layfield L J, Ehya H, Filie A C (2014). Utilization of ancillary studies in the cytologic diagnosis of biliary and pancreatic lesions: The papanicolaou society of cytopathology guidelines for pancreatobiliary cytology. Diagn Cytopathol.

[22] Argani P, Iacobuzio-Donahue C, Ryu B (2001). Mesothelin is overexpressed in the vast majority of ductal adenocarcinomas of the pancreas: identification of a new pancreatic cancer marker by serial analysis of gene expression (SAGE). Clin Cancer Res.

[23] Inaguma S, Wang Z, Lasota J (2017). Comprehensive immunohistochemical study of mesothelin (MSLN) using different monoclonal antibodies 5B2 and MN-1 in 1562 tumors with evaluation of its prognostic value in malignant pleural mesothelioma. Oncotarget.

[24] Jhala N, Jhala D, Vickers SM (2006). Biomarkers in diagnosis of pancreatic carcinoma in fine-needle aspirates: a translational research application. Am J Clin Pathol.

[25] Morello A, Sadelain M, Adusumilli PS (2016). Mesothelin-targeted CARs: driving T cells to solid tumors. Cancer Discov.

[26] Einama T, Homma S, Kamachi H (2012). Luminal membrane expression of mesothelin is a prominent poor prognostic factor for gastric cancer. Br J Cancer.

[27] Zhang J, Khanna S, Jiang Q (2017). Efficacy of anti-mesothelin immunotoxin RG7787 plus nab-paclitaxel against mesothelioma patient-ferived xenografts and mesothelin as a biomarker of tumor response. Clin Cancer Res.

[28] Jiang H, Song B, Wang P (2017). Efficient growth suppression in pancreatic cancer PDX model by fully human anti-mesothelin CAR-T cells. Protein Cell.

[29] Stromnes IM, Schmitt TM, Hulbert A (2015). T cells engineered against a native antigen can surmount immunologic and physical barriers to treat pancreatic ductal adenocarcinoma. Cancer Cell.

[30] June CH (2007). Adoptive T cell therapy for cancer in the clinic. J Clin Invest.

[31] Carpenito C, Milone MC, Hassan R (2009). Control of large, established tumor xenografts with genetically retargeted human T cells containing CD28 and CD137 domains. Proc Natl Acad Sci USA.

[32] Gross G, Waks T, Eshhar Z (1989). Expression of immunoglobulin-T-cell receptor chimeric molecules as functional receptors with antibody-type specificity. Proc Natl Acad Sci USA.

[33] Murad JM, Graber D, Sentman CL (2018). Advances in the use of natural receptor-or ligand-based chimeric antigen receptors (CARs) in haematologic malignancies. Best Pract Res Clin Haematol.

[34] Sadelain M, Brentjens R, Rivière I (2013). The basic principles of chimeric antigen receptor design. Cancer Discov.

[35] Zhong XS, Matsushita M, Plotkin J (2010). Chimeric antigen receptors combining 4-1BB and CD28 signaling domains augment PI3kinase/AKT/Bcl-XL activation and CD8^+^ T cell-mediated tumor eradication. Mol Ther.

[36] Grupp SA, Kalos M, Barrett D (2013). Chimeric antigen receptor-modified T cells for acute lymphoid leukemia. N Eng J Med.

[37] Kochenderfer JN, Dudley ME, Kassim SH (2015). Chemotherapy-refractory diffuse large B-cell lymphoma and indolent B-cell malignancies can be effectively treated with autologous T cells expressing an anti-CD19 chimeric antigen receptor. J Clin Oncol.

[38] Hassan R, Ebel W, Routhier EL (2007). Preclinical evaluation of MORAb-009, a chimeric antibody targeting tumor-associated mesothelin. Cancer Immun.

[39] Sadelain M (2015). CAR therapy: the CD19 paradigm. J Clin Invest.

